# Pharmacologic Stem Cell Based Intervention as a New Approach to Osteoporosis Treatment in Rodents

**DOI:** 10.1371/journal.pone.0002615

**Published:** 2008-07-09

**Authors:** Takayoshi Yamaza, Yasuo Miura, Yanming Bi, Yongzhong Liu, Kentaro Akiyama, Wataru Sonoyama, Voymesh Patel, Silvio Gutkind, Marian Young, Stan Gronthos, Anh Le, Cun-Yu Wang, WanJun Chen, Songtao Shi

**Affiliations:** 1 Center for Craniofacial Molecular Biology, University of Southern California School of Dentistry, Los Angeles, California, United States of America; 2 Graduate School of Medicine, Kyoto University, Kyoto, Japan; 3 National Institute of Dental and Craniofacial Research, National Institutes of Health, Bethesda, Maryland, United States of America; 4 Mesenchymal Stem Cell Group, Division of Haematology, Institute of Medical and Veterinary Science, Adelaide, South Australia, Australia; 5 Division of Oral Biology & Medicine, University of California Los Angeles School of Dentistry, Los Angeles, California, United States of America; Baylor College of Medicine, United States of America

## Abstract

**Background:**

Osteoporosis is the most prevalent skeletal disorder, characterized by a low bone mineral density (BMD) and bone structural deterioration, leading to bone fragility fractures. Accelerated bone resorption by osteoclasts has been established as a principal mechanism in osteoporosis. However, recent experimental evidences suggest that inappropriate apoptosis of osteoblasts/osteocytes accounts for, at least in part, the imbalance in bone remodeling as occurs in osteoporosis. The aim of this study is to examine whether aspirin, which has been reported as an effective drug improving bone mineral density in human epidemiology studies, regulates the balance between bone resorption and bone formation at stem cell levels.

**Methods and Findings:**

We found that T cell-mediated bone marrow mesenchymal stem cell (BMMSC) impairment plays a crucial role in ovariectomized-induced osteoporosis. *Ex vivo* mechanistic studies revealed that T cell-mediated BMMSC impairment was mainly attributed to the apoptosis of BMMSCs via the Fas/Fas ligand pathway. To explore potential of using pharmacologic stem cell based intervention as an approach for osteoporosis treatment, we selected ovariectomy (OVX)-induced ostoeporosis mouse model to examine feasibility and mechanism of aspirin-mediated therapy for osteoporosis. We found that aspirin can inhibit T cell activation and Fas ligand induced BMMSC apoptosis *in vitro*. Further, we revealed that aspirin increases osteogenesis of BMMSCs by aiming at telomerase activity and inhibits osteoclast activity in OVX mice, leading to ameliorating bone density.

**Conclusion:**

Our findings have revealed a novel osteoporosis mechanism in which activated T cells induce BMMSC apoptosis via Fas/Fas ligand pathway and suggested that pharmacologic stem cell based intervention by aspirin may be a new alternative in osteoporosis treatment including activated osteoblasts and inhibited osteoclasts.

## Introduction

Osteoporosis is the most prevalent skeletal disorder, characterized by a low bone mineral density (BMD) and structural deterioration of bone tissue, leading to bone fragility fractures [1]. Postmenopausal osteoporosis is the most common and significant form of osteoporosis in which estrogen deficiency gives rise to a high turnover rate in bone metabolism, as characterized by a over-activated osteoclast activity and a temporal increase in osteoblast activity that is not able to balance osteoclast-mediated bone resorption [1]. Although many systemic and local regulators are involved in estrogen-deficient osteoporosis, it appears that activated T lymphocyte associated osteoclast over-activation play a pivotal role in postmenopausal osteoporosis [2]–[4]. Clinically, bisphosphonates are widely used and appear to ameliorate the effects of osteoporosis by inhibiting osteoclast functions. However, recent experimental evidences suggest that bisphosphonates may also inhibit apoptosis of both osteocytes and osteoblasts [5] and increase the bone forming potential of osteoblasts [6], implying that BMMSC/osteoblast lineage cells may actively participate in the recovery of osteoporosis.

BMMSCs are multipotent postnatal stem cells that are capable of differentiating into osteoblasts, chondrocytes, adipocytes, cardiomyocytes, and myoblasts [7]–[9]. It is known that osteoblasts form new bone matrix to balance osteoclast-mediated bone resorption during the bone remodeling process so as to maintaining homeostasis of the bone/marrow system [10]. The interplay between the BMMSC/osteoblast lineage and hematopoietic stem cells (HSCs) has been reported to play a critical role in regulating the immune system [11]. For instance, culture-expanded BMMSCs are capable of treating T-cell-associated disorders, such as acute graft-versus-host-disease (GVHD) through inhibiting T lymphocyte proliferation and activity [11]–[13]. A recent convergence of clinical and basic research has highlighted a potential link between osteogenic cell death and osteoporosis [14], [15]. It has been suggested that inappropriate apoptosis of osteoblasts/osteocytes accounts for, at least in part, the imbalance in bone remodeling as occurs in osteoporosis, although the elevated activity of osteoclasts is currently thought to be a dominant factor [16], [17].

The objective of this study was to investigate the effects of activated T cells on BMMSCs in the OVX-induced osteoporosis and examine whether aspirin, which has been identified as an effective drug capable of improving bone mineral density in human epidemiological studies [18], [19], cures the OVX-induced osteoporosis via regulating BMMSC function.

## Materials and Methods

### Antibodies

Rat anti-mouse CD3 IgG_3_, anti-mouse CD25 IgM, FITC- or PE-conjugated rat anti-mouse CD25 IgG_1_, FITC-, PE- or PerCP-conjugated rat anti-mouse CD4 IgG_2a_, and PE-conjugated rat anti-mouse CD45R/B220 IgG_2a_ were purchased from BD Bioscience. Rabbit anti-human Runx2 IgG was from Oncogene. Rabbit anti-human alkaline phosphatase (ALP) (LF47) and anti-human osteocalcin (OCN) (LF32) were kindly provided by Dr. Larry Fisher (National Institute of Dental and Craniofacial Research, National Institutes of Health). Mouse anti-human β-actin IgG was purchased from Sigma-Aldrich Co.

### Animals

C3H and C3MRL-Fas*^lpr^* mice were purchased from Jackson Lab. The generation of ovariectomy (OVX) mice was performed the same as described previously [20]. Anti-CD25 antibody (1 mg/mouse, R&D system) was injected intraperitoneally into 3-month-old mice at 2 days prior to the OVX procedure. CD4^+^CD25^−^CD45RB^+hi^ (1×10^6^) and CD4^+^CD25^−^CD45RB^−/low^ (1×10^6^) T cells isolated from spleen of C3H mice by flow cytometry were injected into the tail vein of immunocompromised mice (bg-nu/nu-xid, Harlan Sprague Dawley Inc.). Aspirin (0.6 mg/ml, Sigma-Aldrich Co) dissolved in water to feed mice for two months before OVX procedure. These animal experiments were performed under an institutionally approved protocol for the use of animal research (USC #10874 and NIDCR #03-277).

### Mouse Bone Marrow Mesenchymal Stem cell (BMMSC) culture

Mouse bone marrow cells (10–20×10^6^) harvested from long bones were seeded into 100 mm culture dishes (Corning Costar Co.), incubated for 3 hours at 37°C to allow attachment of adherent cells, and then rinsed twice with PBS to remove the non-adherent cells. Bone marrow cells (10–20×10^6^) from long bones of guinea pigs were then added into each dish as feeder cells. To prevent proliferation in culture, the feeder cells were γ-irradiated (Caesium-137) with 6,000 cGy by a Gammacell-1000 Irradiator (Atomic Energy of Canada. Ltd.). BMMSCs formed adherent colonies following 12–15 days culture. Primary cultures were passed to disperse the colony-forming cells (passage 1). The cells at passage 1 at ∼70% confluence were utilized for the experiments. Culture medium consisted of α-MEM (Invitrogen Corp.), 20% fetal bovine serum (FBS; Equitech-Bio Inc.), 2 mM L-glutamine, 100 U/ml penicillin / 100 µg/ml streptomycin (Biofluids Inc), and 55 µM 2-mercaptoethanol (2-ME). For the osteogenic induction *in vitro*, 2 mM β-glycerophosphate (Sigma-Aldrich Co.), 100 µM L-ascorbic acid 2-phosphate (Wako Pure Chemical Industries Ltd.) and 10 nM dexamethasone (Sigma-Aldrich Co.) were added into the culture for 6 weeks.

### Human BMMSC culture

Human bone marrow aspirates from healthy human adult volunteers (20–35 years of age) were purchased from AllCells LLC and cultured as reported previously [21]. Single cell suspensions (1–100×10^3^/well) of BMMSCs were cultured in 6-well plates (Corning Costar Co.) with α ~-MEM supplemented with 15% FBS, 100 µM L-ascorbic acid 2-phosphate, 2 mM L-glutamine, and 100 U/ml penicillin / 100 µg/ml streptomycin. After aspirin was added at 2.5 µg/ml and 50 g/ml for 3–7 days, BMMSCs were induced for osteogenic differentiation with additional 1.8 mM KH_2_PO_4_ and then harvested for Western blot analysis.

### Isolation and culture of lymph node (LN) and spleen cells

Lymph nodes were collected from axillary and submandibular regions of mice. The LN cells (1.0–2.0×10^6^) were cultured with or without plate-bounded 1 µg/ml anti-CD3 antibody and 1 µg/ml soluble anti-CD28 antibody (BD Bioscience) in complete DMEM containing 10% FBS for 3 days. The culture supernatant was collected and enrichment 10 times to use as a conditioned media (CM).

### Co-culture mouse BMMSCs with LN cells

Mouse BMMSCs (200×10^3^/well) were plated in 24-well flat-bottom plates (Corning Costar Co.), and cultured in mouse BMMSC medium for 3 days. Mouse LN cells isolated from C3H or immunocompromised mice (1×10^6^/well) were loaded directly on mouse BMMSC culture or separated from mouse BMMSCs using Transwell system (Corning Costar Co.). The co-culture systems were cultured for 3–5 days in DMEM medium. Some of the co-cultures were treated with 1 µg/ml anti-interferon γ (IFNγ) antibody, 1 µg/ml anti-tumor necrosis factor α (TNFα) antibody, 1 µg/ml anti-Fas ligand (FasL) antibody (eBioscience or Biolegend), 10 mM brefeldin A (Sigma-Aldrich Co.), 100 nM concanamycin A (Sigma-Aldrich Co.). The cultures were washed to remove LN cells and stained with solution containing 2% paraformaldehyde and 2% toluidine blue or by using an ApopTag® Peroxidase In Situ Apoptosis Detection kit (Chemicon International, Millipore) to detect apoptotic cells.

### Cell viability assay

Cell viability was assayed using Cell Counting Kit-8 (Dojindo) according to the manufacturer's protocol.

### Transplantation of BMMSCs into immunocompromised mice

Approximately 4.0×10^6^ of mouse BMMSCs were mixed with 40 mg of hydroxyapatite/tricalcium phosphate (HA/TCP) ceramic powder (Zimmer Inc.) and then transplanted into the dorsal surface of 10-week-old immunocompromised mice as previously described [20], [21]. These procedures were performed in accordance to specifications of an approved small animal protocol (USC#10874). The transplants harvested at 8 weeks post-transplantation were fixed in 4% paraformaldehyde and then decalcified with 10% EDTA (pH 8.0) for paraffin embedding. Paraffin sections were deparaffinized, rehydrated, and stained with hematoxylin and eosin (H&E). For quantification of new bone regeneration in vivo, the NIH image software Image J was used as previously described [20].

### Bone analyses by micro-computed tomography (microCT) and peripheral quantitative CT (pQCT)

Distal femoral metaphyses were analyzed by microCT (μCT-20; SCANCO USA, Inc.). Scanning regions were confined to secondary spongiosa and were ∼0.30 mm in thickness. Using 2-dimensional images, a region of interest was manually drawn near the endocortical surface. Cancellous bone morphometric indices, assessed using 3-dimensional image reconstructions, included bone volume relative to tissue volume (BV/TV, %), trabecular thickness (Tb.Th), trabecular number (Tb.N) and trabecular separation (Tb.Sp). pQCT analysis of the distal femora was performed using a XCT Research M (Stratec; Norland Co.) as previously described [20]. Briefly, scans were obtained at 2.25 and 2.75 mm from the distal condyles and cancellous BMD. Machine cancellous BMD precision (based on manufacturer data) is ±3 mg/cm^3^ while the coefficient of variation in our laboratory based on repeat scans was 2.26%.

### Colony forming Unit fibroblasts (CFU-F) assay

The CFU-F assay was performed as previously described [20]. Cell aggregates containing more than 50 cells were counted as colonies using a dissecting microscope. The CFU-F assay was repeated in 5 independent experiments.

### Cell proliferation assay

The proliferation of BMMSCs was assessed by bromodeoxyuridine (BrdU) incorporation of the cells by using a BrdU staining kit (Invitrogen Co.) as previously described [20]. To quantify cell proliferation capacity of the cells, ten representative images were used to BrdU-positive cell number. Cell proliferation was shown as a percentage of BrdU-positive cells over total nucleated cells.

### *In vitro* mineralization assay

After 6 weeks culture of BMMSCs under osteogenic inductive condition, calcium deposits were detected by staining with 1% alizarin red (Sigma-Aldrich Co.). The mineralized area were quantified by using NIH image Image-J, and shown as a percentage of alizarin red-positive area over total area as previously described [20].

### Flowcytometric analysis

Cells isolated from mouse blood or spleen were incubated with 1 µg of FITC- or PE-conjugated mAbs for 45 min at 4°C. Isotype-matched FITC- or PE-conjugated IgG were used as controls. After being washed with PBS/0.4%BSA for 3 times, the cells were analyzed by FACS^calibur^ (Becton Dickinson) for analysis. CD4-positive cells were also sorted before flowcytometric analysis.

### Telomerase activity

Telomerase activity in BMMSCs was detected by using a quantitative telomerase detection (QTD) kit (Allied Biotech, Inc.) according to the manufactures' protocol for real-time polymerase chain reaction (PCR) detection. Briefly, cell extraction was prepared from human BMMSCs (100×10^3^), mixed with 2×QTD pre-mix containing telomere primers (TTAGGG) and iQ SYBR green supermix (BioRad Laboratories), and amplified with an iCycler iQ real-time PCR machine (BioRad Laboratories). The generated PCR products are directly detected by measuring the increase in fluorescence caused by binding of SYBR Green to double-strand DNA and calculated by using an iCycler iQ software (BioRad Laboratories). Some cell extract was heated at 85°C for 10 min and used for negative control. The real-time PCR condition was as follows; telomerase reaction for 20 min at 25°C, PCR initial activation step for 3 min at 95°C, 3-step cycling; denaturation for 10 sec at 95°C, annealing for 30 sec at 60°C, extension for 3 min at 72°C, and cycle number 40 cycles.

### Measurement of telomere length

Telomere length of human BMMSCs was measured using Telo*TAGGG* Telomerer Length Assay kit (Rosch, Inc.) according to the manufacturer's protocol. Briefly, genomic DNA was isolated from aspirin (0, 2.5, 50 µg/mL) treated human BMMSCs, enzyme digested and separated on 0.8% agarose gel. Blotting membrane was hybridized with DIG probe followed by anti- DIG-AP and substrate buffer solution, and exposed to X-ray film (Eastman Kodak Co.). NIH image software was used for image analysis. The mean of telomere length was calculated according to manufacturer's instructions

### Western blot analysis

Cells were lysed in M-PER extraction reagent (Pierce Chemical Co.) and protein concentrations were measured using Bio-Rad Protein Assay (Bio-Rad Laboratories Inc.). Ten micrograms of protein were applied to each lane and separated on Tris-Glycine SDS-PAGE gel (Novex; Invitrogen Co.). The proteins were then transferred onto BA-S 85 nitrocellulose membranes (Schleicher & Schuell BioScience Inc,) and blocked for 3 hours at room temperature in NAP blocker (Geno Technology, Inc.). Antibodies used for Western blot included: rabbit anti-alkaline phosphatase (ALP, LF-47), osteocalcin (OCN, LF-32), runt-related transcription factor 2 (Runx 2), β-catenin (Cell Signaling Technology Inc., MA), phospho-β-catenin (Cell signaling Technology inc.), and mouse anti-β-actin antibody. Membranes were incubated with the first antibodies (1∶100–1000 dilution) for 1 hour at room temperature and then incubated with HRP-conjugated secondary antibodies (Santa Cruz Biotechnology, Inc.) at 1∶5000 dilution for 1 hour at room temperature. Following immunolabeling, membranes were washed and reacted with Super Signal chemiluminescence HRP substrate (Pierce Chemical Co.) and then visualized on Kodak X-Omat film (Eastman Kodak Co.).

### Measurement of receptor activator of NFκB ligand (RANKL), osteoprotegerin (OPG) and C-terminal telopeptides of type I collagen

The blood was collected from retro-orbital venous plexus. Enzyme-linked immunoassay (ELISA) was performed to detect RANKL, OPG and C-terminal telopeptides of type I collagen in the blood serum by using commercial kits for mTRANS/RANKL/TNFSF11 Quantikine ELISA, mouse Osteoprotegerin/TNFRSF11B Quantikine ELISA Kits (R&D Systems) and for RatLaps® ELISA kit (Nordic Bioscience Diagnostics A/S), respectively, according to their manufactures' instructions.

### Bone analyses by histology

Femurs and Tibiae were fixed with 4% paraformaldehyde, decalcified with 10% EDTA (pH 8.0) and embedded in paraffin. Sections were deparaffinized and stained with hematoxylin and eosin (H&E). Tartrate-resistant acid phosphate (TRAP) staining was performed according to the previous report [22]. For quantification of TRAP-positive cells in the bones, five representative images were analyzed by using an NIH Image-J. The results were shown as the number of TRAP-positive cells per total bone area.

### *In vitro* osteoclast assay

Pre-osteoblasts were isolated from calvariae of 3-day-old mice by sequential digestion with 3 mg/ml collagenase type I (Worthington Biochemical Co.) and 4 mg/ml dispase (Boehringer Mannheim). Bone marrow cells (200×10^3^/well) or spleen cells (500×10^3^/well) were co-cultured with pre-osteoblasts (10×10^3^/well) under the stimulation of 10 nM 1α, 25(OH)_2_ vitamin D_3_ (BIOMOL Research Laboratories, Inc.) in the 24-well culture plates (Corning Costar Co.) for 7 days. One day after spleen cells (1×10^6^/well) were seeded, 10 ng/ml macrophage colony stimulating factor (R&D Systems) and 10 ng/ml RANKL (Peprotech) were added to the culture for 5–6 days. After TRAP staining, TRAP-positive multinucleated cells (>3 nuclei) were counted as osteoclast-like cells.

### Statistical analysis

Student's *t*-test was used to analyze significance between 2 groups. A P value of less than 0.05 was considered as a significant difference.

## Results

### Activated T lymphocytes induced BMMSC apoptosis through the Fas/FasL pathway

**Figure 1:**
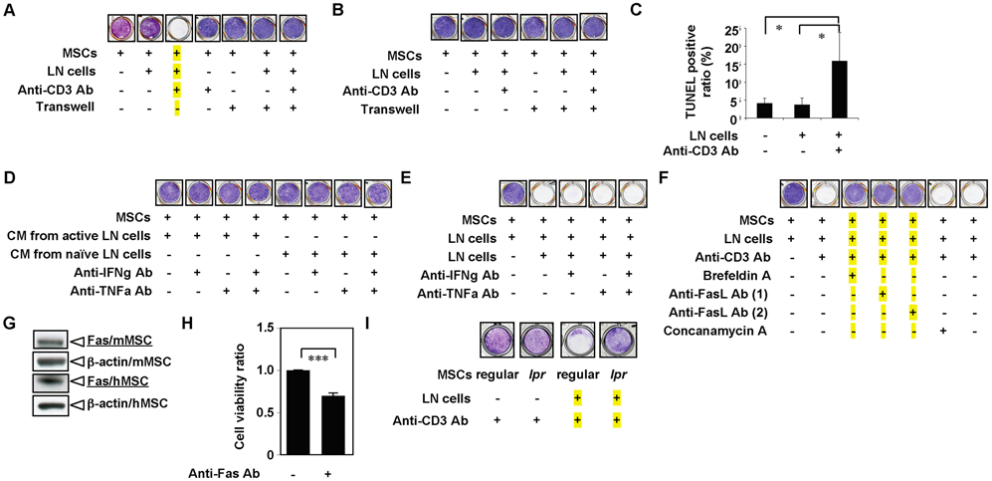
Lymph node (LN) cells activated by an anti-CD3 antibody induce BMMSC apoptosis *in vitro*. (A) Mouse BMMSCs co-cultured with LN cells indicated that anti-CD3 antibody activated LN cells were capable of inducing BMMSC death as shown a blank well without BMMSC staining (blue). When co-cultured BMMSCs (MSC) and LN cells were separated by a transwell culture system, anti-CD3 antibody treated LN cells failed to induce BMMSC death. (B) It is known that immunocompromised mice have no T lymphocytes. Thus, LN cells derived from immunocompromised failed to induce BMMSC death following anti-CD3 antibody activation in the co-culture system. (C) TUNEL staining showed that BMMSC death caused by anti-CD3 antibody-activated LN cell is through an apoptotic pathway. (D) Condition medium (CM) derived from naïve LN cells and anti-CD3 antibody activated LN cells were not able to induce cell death of BMMSCs. (E) Neutralizing anti-TNF-α and IFN-γ antibodies were not able to inhibit BMMSC death induced by anti-CD3 antibody-activated LN cells. (F) Neutralizing Fas ligand antibodies and brefeldin A, but not concanamycin A, were capable of blocking BMMSC death induced by anti-CD3 antibody-activated LN cells. (G) Western blot analysis showed that mouse and human BMMSCs (mMSC and hMSC) express Fas. (H) Fas antibody can induce significant reduction in number of living BMMSCs in culture. (I) Anti-CD3 antibody-activated LN cells were not able to induce cell death of BMMSCs derived from *CD95*-deficient mice (*lpr*). (n = 5; [*P*<0.05 and [[[*P*<0.005).

To determine whether activated T cells directly impinge on BMMSC survival, we used a co-culture system including autologous LN T cells and BMMSCs to assess the effect of T lymphocytes on BMMSCs. When co-cultured with *ex vivo* expanded BMMSCs, the naïve LN cells were unable to induce BMMSC death *in vitro*. However, when LN cells were pre-activated with an anti-CD3 specific antibody, significant amount of cell death occurred in the co-cultured BMMSCs within 12 hours (Fig. 1A). The LN T cell-induced cell death of BMMSCs required direct cell-cell contact, as BMMSCs co-cultured with T cells in the transwell system failed to manifest detectable cell death (Fig. 1A). To further confirm that observed BMMSC death was attributed to T cells in LN population, we treated BMMSCs with anti-CD3 antibody pretreated LN cells derived from T cell deficient immunocompromised mice in the same co-culture conditions, and found no significant induction of BMMSC death (Fig. 1B). Next, we verified that the cell death of BMMSCs was due to apoptosis based on terminal deoxynucleotidyl transferase-mediated dUTP-biotin nick end labeling (TUNEL) staining (Fig. 1C) and 4′,6′-diamidino-2-phenylindole (DAPI) staining (data not shown). These data indicated that activated T cells were required to induce BMMSC death. Conversely, conditioned supernatants collected from anti-CD3 antibody treated LN cell cultures did not cause significant BMMSC death (Fig. 1D), eliminating possibility that soluble factor(s) produced by activated T cells are contributory to cell death of BMMSCs. In support of these observations, inclusion of anti-TNFα and anti- IFN-γ neutralized antibodies in the co-cultures failed to prevent and reverse activated T cell-mediated apoptosis of BMMCs, suggesting that TNF-α and IFN-γ were dispensable (Fig. 1E). Significantly, blockage of Fas-FasL death pathway with either anti-FasL antibodies or a chemical inhibitor, brefeldin A, abrogated LN cell-induced cell death of BMMSCs, indicating the Fas/FasL pathway was critical in the apoptosis of BMMSCs. However, concanamycin A failed to block the activated T cell-induced cell death of BMMSCs (Fig. 1F), suggesting that the perforine/granzyme pathway was not involved. Indeed, we also found that BMMSCs expressed Fas as determined by Western blot analysis (Fig. 1G) and agonistic anti-Fas antibody was capable of inducing apoptosis of human BMMSCs *in vitro* (Fig. 1H). Also, anti-CD3 antibody stimulated spleen cells were capable of inducing apoptosis of BMMSCs and osteoblast-like MG63 cells (data not shown). Finally, we verified that activated T cells failed to induce apoptosis of BMMSCs derived from *CD95*-deficient (*lpr/lpr*) mice (Fig. 1I).

### CD4^+^CD25^−^CD45RB^+hi^ T cell infusion resulted in BMMSC impairment in OVX immunocompromised mice

**Figure 2:**
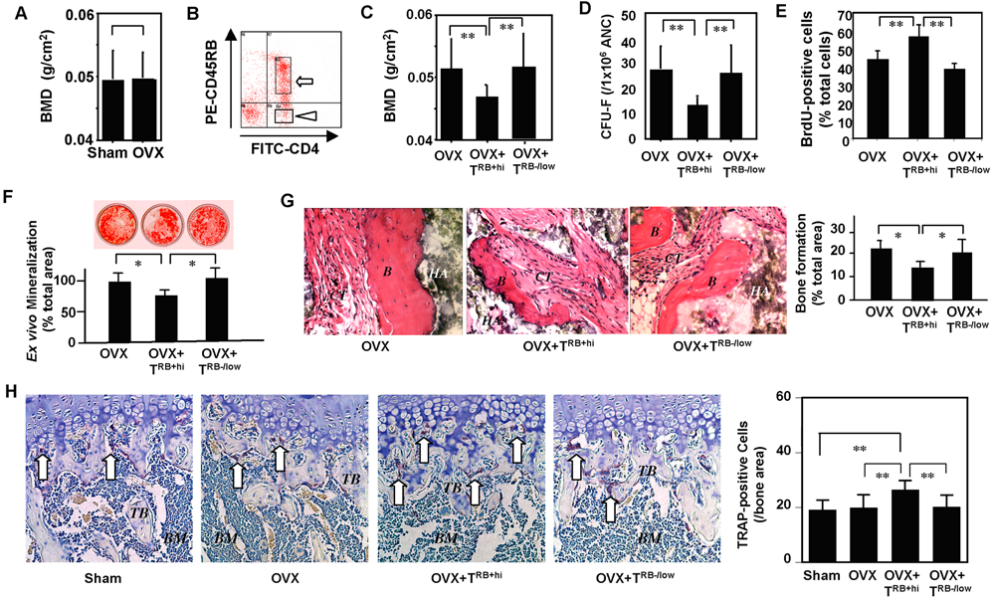
Adoptive transfer of CD4^+^CD25^−^CD45RB^+hi^ T cells derived from C3H mice restores bone loss phenotype in OVX-immunocompromised mice. (A) Estrogen-deficiency failed to induce osteoporosis in 15-week-old bg-nu/nu-xid immunocompromised mice. At one-month post-OVX, there is no BMD difference in femurs between OVX and sham group (n = 4), assessed by Dual x-ray absorptiometry (DEXA) analysis. The graph represents mean±SD. (B) Isolation of CD4^+^CD25^−^CD45RB^+hi^ (open arrow) and CD4^+^CD25^−^CD45RB^−/low^ (triangle arrow) T cells from spleen of C3H mice by flow cytometry for systemic infusion. (C) At one month post-OVX, DEXA analysis revealed a significant decreased BMD in the femurs of CD4^+^CD25^−^CD45RB^+hi^ OVX mice (OVX+T^RB+hi^) when compared to the OVX mice (OVX) or CD4^+^CD25^−^CD45RB^−/low^ OVX mice (OVX+T^RB−/low^; n = 4; [[*P*<0.01). (D) The number of CFU-F decreased significantly in CD4^+^CD25^−^CD45RB^+hi^ OVX mice (OVX+T^RB+hi^) compared to OVX and CD4^+^CD25^−^CD45RB^−/low^ OVX mice (OVX+T^RB−/low^). Totally 10^6^ all nuclear cells (ANC) were used in each group. Error bars represent the mean±SD (n = 5; [[*P*<0.01). (E) The proliferation of BMMSCs from CD4^+^CD25^−^CD45RB^+hi^ OVX mice (OVX+T^RB+hi^) was significantly increased in compared to CD4^+^CD25^−^CD45RB^−/low^ OVX mice (OVX+T^RB−/low^) and OVX mice as assessed by BrdU incorporation assay for 24 hours. The number of BrdU-positive cells was indicated as a percentage to the total number of counted BMMSCs and averaged from 5 replicated cultures. Error bars represent the mean±SD (n = 5; [[*P*<0.001). (F) Alizarin red staining showed that BMMSCs derived from CD4^+^CD25^−^CD45RB^+hi^ OVX mice (OVX+T^RB+hi^) had lower calcium accumulation than that of BMMSCs from OVX and CD4^+^CD25^−^CD45RB^−/low^ OVX mice (OVX+T^RB−/low^) when cultured under the osteogenic inductive conditions. The graph represents mean±SD (OVX mice, n = 4; CD4^+^CD45RB^+hi^ OVX mice, n = 5; CD4^+^CD45RB^−/low^ OVX mice, n = 4; [*P*<0.001). (G) BMMSCs were transplanted into immunocompromised mice using HA/TCP (*HA*) as a carrier for eight weeks. Bone formation assessed by H&E staining was decreased in transplants of BMMSCs derived from CD4^+^CD25^−^CD45RB^+hi^ OVX mice (OVX+T^RB+hi^) compared to BMMSC transplants of OVX and CD4^+^CD25^−^CD45RB^−/low^ OVX mice (OVX+T^RB−/low^). *B*; bone *CT*; connective tissue. Original magnification; ×200. The graph represents mean±SD (OVX mice, n = 4; CD4^+^CD45RB^+hi^ OVX mice, n = 5; CD4^+^CD45RB^−/low^ OVX mice, n = 4; [*P*<0.001). (H) TRAP staining showed that 15-week old OVX mice received CD4^+^CD45RB^hi^ cells (OVX+T^RB+hi^) contain elevated number of TRAP-positive osteoclastic cells (white arrows) in epiphysis and trabecular bone areas of femurs as compared to Sham, OVX, and OVX received CD4^+^CD45RB^−/low^ cells (OVX+T^RB−/low^). The graph represents mean±SD (n = 4; [*P*<0.05, [[*P*<0.01). BM: bone marrow, TB: trabecular bone.

To examine whether T cell can impair BMMSC *in vivo*, we adoptively transferred CD4^+^CD25^−^CD45RB^+hi^ T cells, previously showed to induce inflammatory bowel disease (IBD) in nude mice [23] and in patients with IBD associated osteoporosis [24], [25], into OVX immunocompromised mice that lacked osteoporosis phenotype due to the absence of T lymphocytes (Fig. 2A). OVX-immunocompromised mouse is an established model with inducible osteoporosis phenotype that allows testing whether CD4^+^CD25^−^CD45RB^+hi^ T cell can induce osteoporosis like bone density alteration *in vivo*. We isolated CD4^+^CD25^−^CD45RB^+hi^ and CD4^+^CD25^−^CD45RB^−/low^ T cells from the spleens of C3H mice (Fig. 2B) and intravenously infused into immunocompromised mice. One month after the infusion, the typical trabecular bone resorption and lumbar BMD reduction were observed in OVX-immunocompromised mice that received CD4^+^CD25^−^CD45RB^+hi^ T cells, but not CD4^+^CD25^−^CD45RB^−/low^ T cells (Fig. 2C), suggesting that CD4^+^CD25^−^CD45RB^+hi^ T cells were responsible, at least in great part, for T-cell-associated osteoporosis. Next, we assessed whether BMMSC deficiency contributed to the osteoporosis phenotype in immunocompromised mice receiving CD4^+^CD25^−^CD45RB^+hi^ T cells. By examining the CFU-F efficiency, an assay representing the number of clonogenic mesenchymal progenitors, we found a significantly decreased number of CFU-F in CD4^+^CD25^−^CD45RB^+hi^ cell-treated OVX-immunocompromised mice compared to the un-treated or CD4^+^CD25^−^CD45RB^−/low^ T cell-treated OVX-immunocompromised animals (Fig. 2D). In addition, the proliferation of BMMSCs from CD4^+^CD25^−^CD45RB^+hi^ T cell-treated OVX-immunocompromised mice was also dramatically increased compared to the other two groups, as assessed by the BrdU incorporation assay (Fig. 2E). Moreover, we found that the systemic infusion of CD4^+^CD25^−^CD45RB^+hi^ T cells led to an impaired osteogenic differentiation of BMMSCs, as shown by decreased mineralization accumulation in osteogenic inductive cultures (Fig. 2F) and reduced bone formation when transplanted subcutaneously into immunocompromised mice (Fig. 2G). It is known that elevated osteoclast activity plays a major role in osteoporosis. Here we found that the number of TRAP-positive osteoclasts was significantly increased in CD4^+^CD25^−^CD45RB^+hi^ T cell-treated OVX-immunocompromised mice compared to the control groups (Fig. 2H). Taken together, the data indicated that both impaired BMMSCs and activated osteoclast activity in CD4^+^CD25^−^CD45RB^+hi^ T cell-treated OVX-immunocompromised mice contributed to the osteoporosis phenotype.

### Aspirin recovered OVX-induced osteoporosis by improving osteogenesis and inhibiting osteoclastogenesis

**Figure 3:**
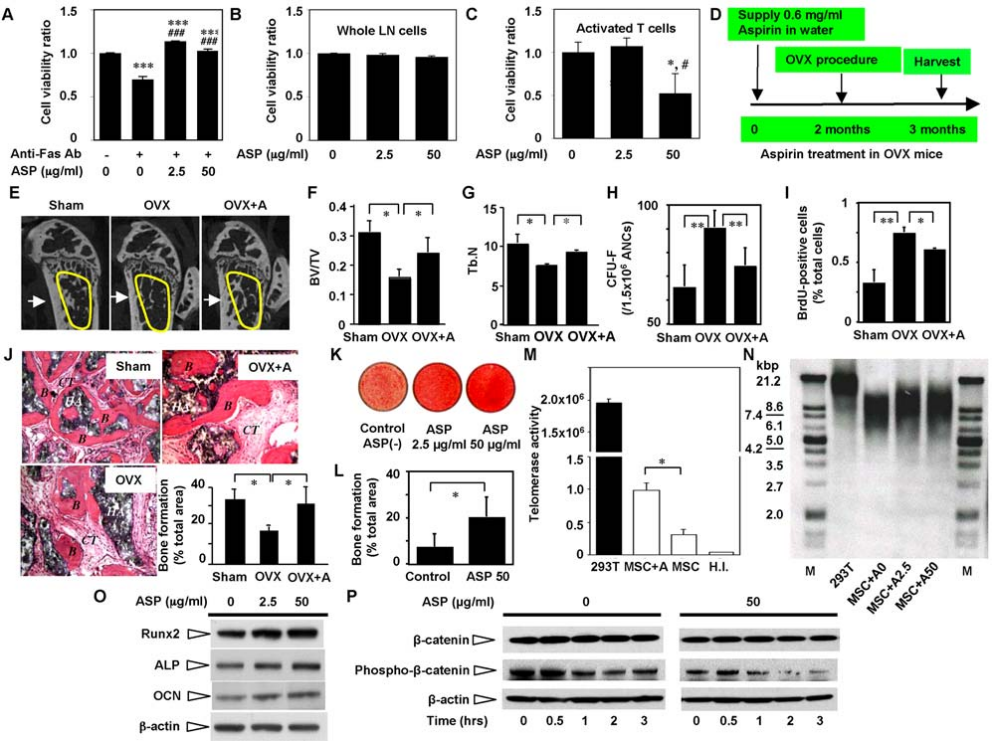
Aspirin treatment induces activated T cell death *in vitro*, promotes osteogenesis of BMMSCs, and improves BMD in OVX mice. (A) Fas Ab can induce significantly reduction in number of living BMMSCs in the culture. However, the cell viability of human BMMSCs was significantly improved in aspirin (ASP)-treated groups (n = 5, [[[*P*<0.005 vs. untreated control group; ###*P*<0.005 vs. Fas Ab-treated group). (B) Aspirin (ASP) did not affect the cell viability of whole LN cells cultured on regular plate. (C) On the other hand, T cells activated by cultured on CD3εΑb-coated dishes showed decreased viability in 50 µg/ml ASP-treated group, but not in 2.5 µg/ml ASP-treated group. (n = 5, [*P*<0.05 vs. 0 µg/ml ASP group; #*P*<0.05 vs. 2.5 µg/ml ASP group). (D) The scheme indicates the experimental design on aspirin administration and OVX surgery procedure. (E) Trabecular bone structure of the distal femoral metaphysis was analyzed by microQCT. As expected OVX mice showed decreased trabecular bone (yellow circle area) and cortical bone (arrow) formation when compared to sham mice. Aspirin treated OVX mice exhibited a significant increase in trabecular bone (yellow circle area) and cortical bone (arrow) volume compared to OVX mice. (F,G) MicroQCT analysis demonstrated that BV/TV (bone volume vs. tissue volume) (F) and Tb.N (trabecular number) (G) were decreased in OVX mice as compared to sham group but significantly increased in aspirin treated OVX group (n = 5; [*P*<0.01). (H) The number of CFU-F in OVX group increased significantly as compared to sham group, however, aspirin treatment (OVX+A) appeared to reduce CFU-F number to the sham group level. Error bars represent the mean±SD (n = 5, [[*P*<0.01). (I) The proliferation rate of BMMSCs was assessed by BrdU incorporation assay for 24 hours. The number of BrdU-positive cells was indicated as a percentage to the total number of counted BMMSCs and averaged from 5 replicated cultures. BMMSCs derived from OVX mice showed significantly elevated BrdU-uptake rate, however, aspirin treatment (OVX+A) reduced BrdU up-take rate. Error bars represent the mean±SD (n = 5, [*P*<0.01, [[*P*<0.001). (J) BMMSCs from aspirin treated mice were transplanted into immunocompromised mice for eight weeks using HA/TCP (*HA*) as a carrier vehicle. Bone formation assessed by H&E staining was decreased in OVX BMMSC transplants compared to sham mouse group. Aspirin treatment (OVX+A) can improve OVX BMMSC-mediated bone formation *in vivo*. *B*; bone *CT*; connective tissue. Original magnification; ×200. The bone formation rate was calculated as the percentage of newly formed bone area per total area of transplant at the representative cross-sections. The graph represents mean±SD (sham mice, n = 4; OVX mice, n = 4; OVX+A mice, n = 4; [*P*<0.01). (K) Representative images of Alizarin red staining of human BMMSCs cultured under the osteogenic inductive condition containing 2.5 and 50 µg/ml aspirin (ASP 2.5 µg/ml and ASP 50 µg/ml). Aspirin treatment can increase calcium accumulation in cultured human BMMSCs. (L) After aspirin treatment at 50 µg/ml for 1 week, *ex vivo* expanded human BMMSCs were transplanted into immunocompromised mice with HA/TCP (*HA*) as a carrier. Aspirin treated human BMMSCs exhibited significantly increased new bone formation in comparison to non-aspirin-treated control human BMMSC transplants ([ *P*<0.05). (M) Human BMMSCs express low levels of telomerase activity (MSC), if there is any. Following 1-week aspirin treatment (2.5 or 50 µg/ml), human BMMSCs showed a significantly increased telomerase activity (MSC+A) ([ *P*<0.01). HEK293 cells were used as a positive control (239T) and heat inactive human BMMSCs were used as negative control (H.I). (N) Aspirin treatment at 2.5 µg/ml (Asp 2.5) and 50 µg/ml (Asp 50) can slightly increased telomere length from 6.4 kb in un-treated control group (Asp 0) to 7.7 kb (Asp 2.5) and 7.6 (Asp 50) respectively. 293T cell line (12.1 kb) was used as a positive control. (O) Western blot analysis showed that aspirin treatment (ASP) at indicated dosages enables to elevate expressions of Runx2, alkaline phosphatase (ALP), and osteocalcin (OCN). β-actin was used a control for the amount of sample loading. (P) Human BMMSCs were cultured with or without aspirin at 50 µg/ml for 0, 0.5, 1, 2, 3 hours. The degradation of phospho-β-catenin was accelerated in aspirin (50 µg/ml) treated group. β-actin was used a control for the amount of sample loading.

**Figure 4:**
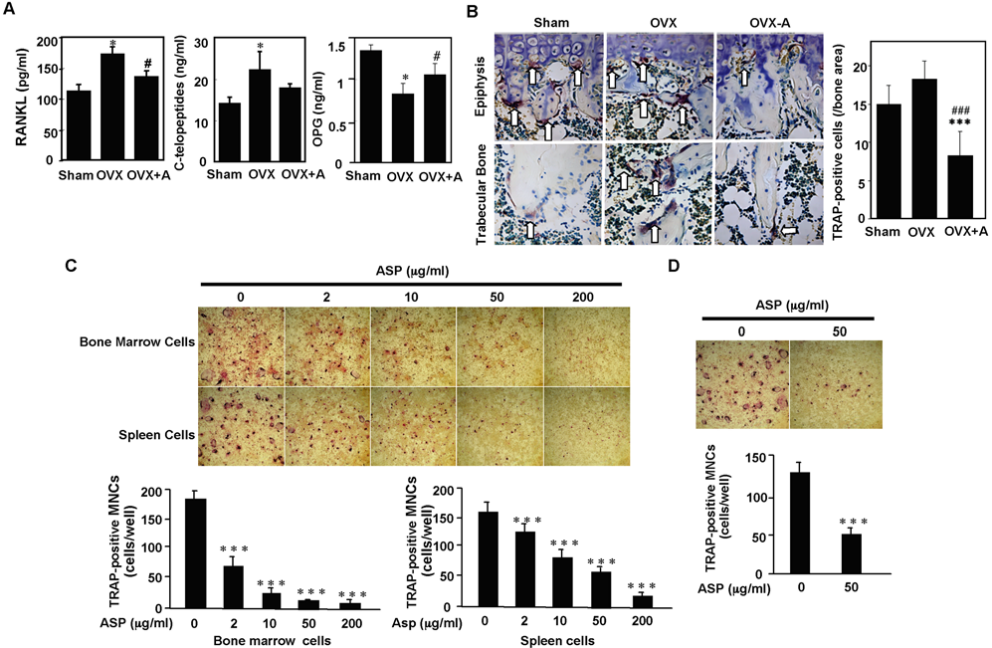
Aspirin treatment inhibits osteoclast activities. (A) OVX mice have increased levels of RANKL and type I collagen C-terminal telopeptides and decreased levels of OPG in blood serum as compared to sham mice. Aspirin treated OVX mice (OVX+A) showed a significant decreased levels of RANKL and type I collagen C-terminal telopeptides along with increased levels OPG in blood serum. The graph represents mean±SD (n = 5; [*P*<0.01 vs. Sham; #*P*<0.05 vs. OVX). (B) TRAP staining confirmed that OVX mice had increased number of TRAP positive cells in epiphysis and trabecular bone areas of the distal femurs as compared to sham mice. Administration of aspirin for three months resulted in a significant decrease in number of TRAP positive cells (OVX+A) in the epiphysis and trabecular bone areas (n = 6; [[[*P*<0.005 vs. Sham; ###*P*<0.005 vs. OVX). (C) *Ex vivo* co-culture bone marrow cells or spleen cells with osteoblastic cells revealed that *in vitro* aspirin treatment inhibits the formation of TRAP-positive multinuclear cells (MNCs) in a dose dependent manner (2–200 µg/ml). The graph represents mean±SD (n = 5; [[[*P*<0.005 vs. ASP 0 µg/mL). (D) RANKL-induced osteoclastogenesis was partially blocked by aspirin treatment at 50 µg/ml as seen a significantly decreased number of TRAP-positive osteoclasts in aspirin treated group (ASP 50). The graph represents mean±SD (n = 5; [[[*P*<0.005 vs. ASP 0 µg/mL).

It was known that aspirin improves bone density in aged population [18], [19], therefore, we examined whether aspirin affects Fas-induced BMMSC apoptosis and found that indeed aspirin is capable of inhibiting Fas antibody-induced cell death of BMMSCs (Fig. 3A) and specifically inducing death of activated T cells (Fig. 3B and 3C). To elucidate the role of aspirin in osteoporosis treatment, we used OVX mice treated with low dose aspirin for three months (Fig 3D). Using microQCT analysis we found that aspirin treatment significantly improved trabecular and cortical bone density in OVX mice (Figs. 3E–3G). In addition, aspirin treatment suppressed OVX-induced high number of CFU-F and proliferation rate of BMMSCs (Figs. 3H and 3I). When BMMSCs were transplanted into immunocompromised, the aspirin treated mice showed elevated bone-forming capacity (Fig. 3J). To further examine the mechanism of aspirin-mediated improvement of osteogenesis, we revealed that aspirin treatment was capable of promoting mineral accumulation of human BMMSCs in osteo-inductive cultures (Fig. 3K) and significantly increased bone formation when these BMMSCs were subcutaneously transplanted into immunocompromised mice (Fig. 3L). Previously, it was reported that telomerase activation in BMMSCs improves osteogenesis and adipogenesis [21]. Thus, we examined whether aspirin treatment elevated telomerase activity in BMMSCs as seen in epithelial cells [26]. As expected, we found that aspirin treatment slightly raised telomerase activity in BMMSCs (Fig. 3M) and their telomere lengths (Fig. 3N). The underlying mechanisms of aspirin effect on bone metabolism appear to correlate with increased expression of certain osteogenic genes including Runx2, a master gene for osteogenic differentiation, ALP, and OCN (Fig. 3O). Additionally, *ex vivo* aspirin treatment was capable of accelerating degradation of phospho-β-catenin (Fig. 3P), resulting in an increased level of WNT signaling, a recognized pathway in osteogenesis. Since a balance between bone formation and resorption is required for maintaining bone integrity in the remodeling process. We further examine the role of aspirin on osteoclast activity and found that long–term aspirin treatment can systemically alter several serum markers, reduced RANKL and increased OPG in OVX mice (Fig. 4A). Although we found a decreased level of C-terminal telopeptide in aspirin treated OVX mice, it failed to show a statistically significant difference (Fig. 4A). Histological sections showed a marked decrease in TRAP-positive cells in the tibia of aspirin treated OVX mice in comparison to the OVX and sham mice (Fig. 4B). Further studies using osteoclast progenitors derived from the bone marrow and spleen co-cultured with pre-osteoblasts from calvariae revealed that TRAP-positive multinucleated cells (TRAP^+^-MNCs) significantly decreased in the aspirin-treated group in a dose-dependent manner (Fig. 4C). Additionally, *ex vivo* aspirin treatment can block RANKL-induced osteoclastogenesis (Fig. 4D). Taken together, these data indicated that aspirin is capable of improving bone formation and inhibiting bone resorption through multiple mechanisms.

## Discussion

Aspirin may contribute to multiple biological pathways, such as inhibiting COX2 and COX1, and PG2 activities. This makes it difficult to elucidate the detailed functional mechanism of aspirin in relation to bone remodeling. Epidemiological studies reported that the regular use of aspirin or nonsteroidal anti-inflammatory drugs (NSAIDs) may have a moderate beneficial effect on BMD in postmenopausal woman [18], however, there appears no clinically significant protective effect on the subsequent risk of fractures [19]. This discrepancy may be linked to the multiple functional roles of aspirin where its activity may largely depend on the *in vivo* microenvironment. Moreover, diversified functional roles of aspirin make it unlikely that any single pathway contributes solely to its anti-apoptotic effect on BMMSCs. In this study, we found that aspirin was able to up-regulate telomerase activity as seen in other cell types [26] with capability to increase bone formation [21]. We speculate that up-regulated telomerase activity in BMMSCs resulted in aspirin-mediated osteogenesis, suggesting that aspirin could serve a potential treatment of osteoporosis in the OVX mice. Aspirin-elevated telomerase level in BMMSCs is much lower than that in cancer cells, implying a safe use of aspirin to improve osteogenesis.

Although it has been proposed that anti-osteogenic cell apoptosis or improved osteogenic cell function is an effective approach for osteoporosis treatment [27] as occurs with the application of PTH [28], simultaneously targeting of osteoclasts and osteoblasts to treat osteoporosis may be a more potent therapeutic approach. Despite the fact that well-developed anti-osteoclast drugs, such as bisphosphonates and strontium, have achieved reasonable therapeutic effects in osteoporosis patients [29], [30], their functions may also involve an independent effect on improving osteogenic cell function [6], [31]. More importantly, it is necessary to examine whether this combination therapy is a superior choice for the treatment of osteoporosis than the currently available therapeutic regimen.

It has been reported that the adoptive transfer of T cells can restore OVX-induced bone loss in nude mice [32]. Transfer of CD4^+^CD45RB^+hi^ T cells to immune-deficient recipients leads to the development of IBD [23], [33]. In contrast, transfer of the reciprocal CD4^+^CD45RB^−/low^ population not only failed to induce colitis, but also prevented it [34]. Interestingly, patients with IBD often have decreased bone mass, an increased risk of developing osteoporosis, and associated fragility fractures and morbidity [35], [36]. Although osteoclasts and osteoblasts are suspected to be involved in the low bone mass in IBD patients, the precise mechanisms of action remain unclear, especially whether activated T cells act on osteogenic progenitor cells is currently unknown. Therefore, in this study we applied a widely used T-cell adoptive transfer system in mice to elucidate the association between CD4^+^CD45RB^+hi^ T cells and BMMSCs, adding a potential mechanism that may contribute to the bone phenotype in osteoporosis patients. More importantly, we provided direct evidences to support the notion that interplays between T cells and BMMSCs through Fas/FasL signaling pathway may be critical for pathogenesis of osteoporosis.

The Fas/FasL system is a physiologically important pathway for the regulation of tumor cell proliferation and suppression of immune response. Deregulation of Fas/FasL leads to various severe clinical disorders associated with uncontrolled T cell proliferation, such as organ transplantation graft rejection, systemic lupus erythematosus, Sjogren's syndrome, and lymphoid tumors [37]. FasL was originally discovered on cells of the lymphoid/myeloid lineage, including activated T cells, where it plays an important role in immune homeostasis and T cell- and NK cell-mediated toxicity [38]. Expression of FasL in activated T lymphocytes plays an important role in Fas-mediated tumor killing. It has been reported that osteoblasts express Fas and the FasL/Fas pathway involved in osteogenic cell apoptosis in response to glucocorticoids [39]. Here, we found that BMMSCs are akin to osteoblasts expressing Fas. In our study, activated T lymphocytes can trigger BMMSC apoptosis, predominantly through the FasL/Fas pathway, supporting the notion that Fas antigen is involved in BMMSC death *in vivo* [40]. In contrast, the perforin-based pathway, another major mechanism of cell-mediated cytotoxicity, is not involved in activated T cell-mediated BMMSC apoptosis. In this study, we used LN cells to interact with BMMSCs, where the LN population contains both inflammatory and regulatory T cell populations. Other cell types such as macrophages and dendritic cells (DCs) may participate in T cell activation in our co-culture system. Further studies are needed to determine whether and how each subset of T cell affects BMMSC apoptosis.

In summary, this study revealed that activated T lymphocytes are responsible for the BMMSC apoptosis through the Fas/FasL pathway, resulting in accelerating osteoporosis phenotype in OVX mice. Moreover, we found that pharmacologic regulation of BMMSCs by aspirin may offer a new approach for estrogen-deficient osteoporosis treatment.
